# Supplementary material for: A new class of constitutively active super-enhancers is associated with fast recovery of 3D chromatin loops
Source: BMC Bioinformatics. 2019 Mar 29;20(Suppl 3):127. doi: 10.1186/s12859-019-2646-3 (PMC6439976; doi:10.1186/s12859-019-2646-3)
Supplement: Supplementary file 1 — Figure S1. Ubiquitous activity of super-enhancers defined in one cell/tissue type. 196 super-enhancer domains are called for only one cell/tissue type but showed constitutive H3K27ac signals across cell/tissue types and also located at less than 20% percentile of tau score. The heatmap of background subtracted log2 transformed H3K27ac signals of the domains across cell/tissue types is shown. Figure S2. Hierarchical clustering of samples using H3K27ac signal on super-enhancer domains. Complete-link hierarchical clustering of distance between samples with respect to log2 transformed H3K27ac RPM of SE domains. H1 human embryonic cell line and its derived cell lines are shown in brown and immortalized cell lines are shown in blue. Cancer cell lines are shown in bold text. Figure S3. Functional characterization of super-enhancer domain classes with alternative putative target genes. a, Heatmaps showing log2(RPKM+ 1) value of putative target gene of unique super-enhancer domains (top), non-unique super-enhancer domains (middle), and common super-enhancer domains (bottom). b, Histogram of tau score for putative target gene expression in each class of super-enhancer domains. Low tau score indicates universal expression pattern. Table S1. GO analysis of alternative putative target genes of unique super-enhancer domains. Table S2. GO analysis of alternative putative target genes of common super-enhancer domains. Table S3. Source of H3K27ac and input ChIP-seq reads. (PDF 574 kb) [file 12859_2019_2646_MOESM1_ESM.pdf]

Additional File 1

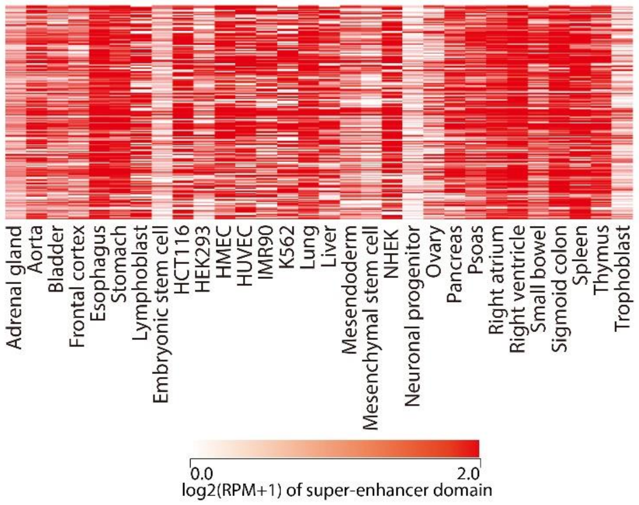

Figure S1. Ubiquitous activity of super-enhancers defined in one cell/tissue type

196 super-enhancer domains are called for only one cell/tissue type but showed constitutive H3K27ac signals across cell/tissue types and also located at less than 20% percentile of tau score. The heatmap of background subtracted log2 transformed H3K27ac signals of the domains across cell/tissue types is shown.

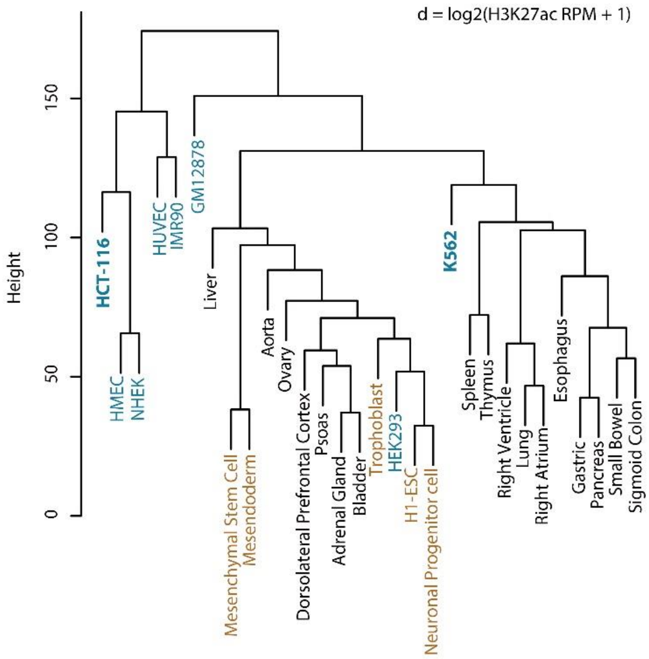

Figure S2. Hierarchical clustering of samples using H3K27ac signal on super-enhancer domains. Complete-link hierarchical clustering of distance between samples with respect to log2 transformed H3K27ac RPM of SE domains. H1 human embryonic cell line and its derived cell lines are shown in brown and immortalized cell lines are shown in blue. Cancer cell lines are shown in bold text.

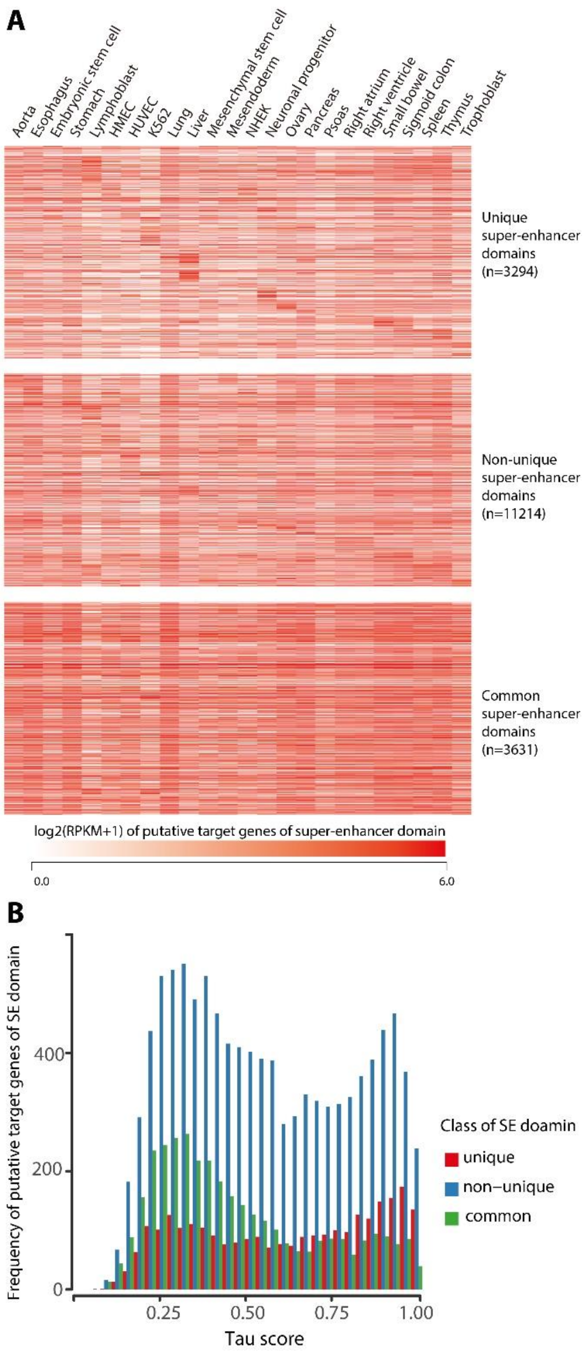

Figure S3. Functional characterization of super-enhancer domain classes with alternative putative target genes

a, Heatmaps showing log2(RPKM+1) value of putative target gene of unique super-enhancer domains (top), non-unique super-enhancer domains (middle), and common super-enhancer domains (bottom). **b,** Histogram of tau score for putative target gene expression in each class of super-enhancer domains. Low tau score indicates universal expression pattern.

Supplementary tables:

Table S1. GO analysis of alternative putative target genes of unique super-enhancer domains

	Term	Fold enrichment
**GM12878 (Lymphoblast)**	GO:0006955~immune response	3.56
	GO:0032729~positive regulation of interferon-gamma production	11.3
	GO:0031295~T cell costimulation	6.68
	GO:0033209~tumor necrosis factor-mediated signaling pathway	4.96
	GO:0006915~apoptotic process	2.30
	GO:0071222~cellular response to lipopolysaccharide	4.61
	GO:0042832~defense response to protozoan	13.7
	GO:0007165~signal transduction	1.74
	GO:0060333~interferon-gamma-mediated signaling pathway	5.50
	GO:0030154~cell differentiation	2.25
	GO:0006954~inflammatory response	2.40
	GO:0045893~positive regulation of transcription, DNA-templated	2.15
	GO:0034138~toll-like receptor 3 signaling pathway	24.4
	GO:0045630~positive regulation of T-helper 2 cell differentiation	24.4
	GO:0040008~regulation of growth	3.52
**Prefrontal Cortex**	GO:0007612~learning	11.6
	GO:0010821~regulation of mitochondrion organization	30.5
	GO:0008299~isoprenoid biosynthetic process	28.3
	GO:0007409~axonogenesis	6.75
	GO:0009240~isopentenyl diphosphate biosynthetic process	132
	GO:0050992~dimethylallyl diphosphate biosynthetic process	132
	GO:0060221~retinal rod cell differentiation	88.1
	GO:0035434~copper ion transmembrane transport	66.1
	GO:0006695~cholesterol biosynthetic process	10.4
	GO:0060291~long-term synaptic potentiation	10.4
	GO:0007010~cytoskeleton organization	4.11
	GO:1901215~negative regulation of neuron death	9.92
	GO:0055069~zinc ion homeostasis	52.8
	GO:0071287~cellular response to manganese ion	44.0
	GO:0009615~response to virus	4.81

Table S2. GO analysis of alternative putative target genes of common super-enhancer domains

Term	Fold Enrichment	p-value
GO:0098609~cell-cell adhesion	2.35	1.92E-12
GO:0000122~negative regulation of transcription from RNA polymerase II promoter	1.62	1.18E-07
GO:0045944~positive regulation of transcription from RNA polymerase II promoter	1.48	1.92E-06
GO:0008360~regulation of cell shape	2.08	4.12E-03
GO:0007179~transforming growth factor beta receptor signaling pathway	2.36	4.66E-03
GO:0030036~actin cytoskeleton organization	2.04	1.54E-02
GO:0048008~platelet-derived growth factor receptor signaling pathway	3.51	1.55E-02
GO:0001666~response to hypoxia	1.85	2.02E-02
GO:0035556~intracellular signal transduction	1.52	2.63E-02
GO:0048870~cell motility	3.68	2.77E-02
GO:0045668~negative regulation of osteoblast differentiation	2.96	3.11E-02

Table S3. Source of H3K27ac and input ChIP-seq reads

	Full Name (Confirmed-181011)	H3K27ac	Input
Tissue	Adrenal gland	ENCFF965NCN	ENCFF072EKO
	Aorta	ENCFF304OFY	ENCFF883ZYF
	Bladder	ENCFF413QKU	ENCFF770IYO
	Dorsolateral Prefrontal Cortex	GSM1866052	GSM1866054
	Esophagus	ENCFF972UCE	ENCFF240RBR
	Stomach	ENCFF669MHN	ENCFF404AFK
	Lung	ENCFF906WKZ	ENCFF587OOH
	Liver	ENCFF007GTB	ENCFF057IKR
	Ovary	ENCFF503FJZ	ENCFF167BJI
	Pancreas	ENCFF996ECL	ENCFF174XJR
	Psoas muscle	ENCFF823MZG	ENCFF411YQM
	Right Atrium	ENCFF812IBZ	ENCFF597CXC
	Right Ventricle	ENCFF004BQX	ENCFF108OEG
	Small intestine	ENCFF308MVS	ENCFF863QDS
	Sigmoid colon	ENCFF375GAE	ENCFF347MJY
	Spleen	ENCFF063UMS	ENCFF182DKW
	Thymus	ENCFF799IYC	ENCFF043CYN
H1-ESC derived cell line	H1-ESC	ENCFF000AWR	ENCFF805KPU
	Mesenchymal stem cell	ENCFF970HHO	ENCFF317PIV
	Mesendoderm	ENCFF296LHG	ENCFF250CGU
	Trophoblast	ENCFF613EXZ	ENCFF312JHP
	Neural Stem Progenitor Cell	ENCFF279KNT	ENCFF591SDP
Immortalized cell line	GM12878	ENCFF000ASU	ENCFF000ARK
	HCT-116	GSM2809617	GSM3242978
	HEK293	ENCFF002ABC	ENCFF000WXY
	HMEC	ENCFF000BIL	ENCFF000BHF
	HUVEC	ENCFF000BSI	ENCFF000BQW
	IMR90	ENCFF200XEH	ENCFF855STX
	K562	ENCFF000BXH	ENCFF000BWK
	NHEK	ENCFF000CNN	ENCFF000CMD
